# The unsolved mystery of apoA-I recycling in adipocyte

**DOI:** 10.1186/s12944-016-0203-x

**Published:** 2016-02-24

**Authors:** Shuai Wang, Dao-quan Peng, Yuhong Yi

**Affiliations:** Department of Cardiology, The Second Xiangya Hospital of Central South University, Changsha, Hunan China; The Second Xiangya Hospital of Central South University, Changsha, Hunan China

**Keywords:** Lipoprotein, Adipocytes, Uptake, Resecretion, Cholesterol

## Abstract

As the major storage site for triglycerides and free cholesterol, adipose tissue plays a central role in energy metabolism. ApoA-I is the main constituent of HDL and plays an important role in removal of excess cholesterol from peripheral tissues. Recently, multiple studies have shown beneficial effects of apoA-I on adipose metabolism and function. ApoA-I was reported to improve insulin sensitivity and exert anti-inflammatory, anti-obesity effect in animal studies. Interestingly, Uptake and resecretion of apoA-I by adipocytes has been detected. However, the significance of apoA-I recycling by adipocytes is still not clear. This article reviewed methods used to study cellular recycling of apoA-I and summarized the current knowledge on the mechanisms involved in apoA-I uptake by adipocytes. Since the main function of apoA-I is to mediate reverse cholesterol transport from peripheral tissues, the role of apoA-I internalization and re-secretion by adipocytes in intracellular cholesterol transport under physiological and pathological conditions were discussed. In addition, findings on the correlation between apoA-I recycling and obesity were discussed. Finally, it was proposed that during intracellular transport, apoA-I-protein complex may acquire cargoes other than lipids and deliver regulatory information when they were resecreted into the plasma. Although apoA-I recycling by adipocytes is still an unsolved mystery, it’s likely that it is more than a redundant pathway especially under pathological conditions.

## Background

Apolipoprotein A-I (apoA-I) is synthesized and secreted by the liver and intestine. As the major protein constituent of high-density lipoprotein (HDL), apoA-I plays an important role in reverse cholesterol transport from peripheral tissues to the liver [1]. As the body’s largest reservoir of free cholesterol, adipose tissue contributes to apoA-I lipidation and nascent HDL biogenesis [2, 3]. On the other side, apoA-I has been reported to have reciprocal effects on adipose tissue metabolism and function. For example, apoA-I has been reported to promote glucose uptake [4], improve insulin sensitivity [5], upregulate the expression of adiponectin [6], and exert anti-inflammatory effect [7, 8]. It is generally accepted that the multiple effects of apoA-I is mediated through regulation of lipid raft or direct binding with surface receptors, resulting in subsequent activation of intracellular signaling [9, 10]. What is interesting is our previous finding that apoA-I protein was found in human adipose tissue while apoA-I mRNA was not detected, indicating that apoA-I found in adipose tissue was exogenous. Actually, cellular internalization of apoA-I has been observed in several cell lines, including macrophages, endothelium and smooth muscle cells [11–13]. However, it is not until recent years that the uptake and resecretion of apoA-I by adipocytes has been confirmed with the significance still remaining unclear [14, 15]. This article briefly discussed methods used to study apoA-I recycling, mechanisms for apoA-I internalization and the possible significance of apoA-I recycling by adipocytes.

### ApoA-I-induced cell signaling and adipocyte metabolism

Except for mediating reverse cholesterol transport, there are several well documented evidence suggesting that apoA-I could regulate cellular function of adipocytes. ApoA-I has been reported to exert anti-inflammatory effect on adipocytes. ApoA-I reduced monocyte chemotactic protein-1 (MCP-1) and serum amyloid A3 (SSA3) expression and attenuated reactive oxygen species (ROS) through diminishing the translocation of NADPH oxidase (nicotinamide adenine dinucleotide phosphate-oxidase) into lipid rafts [7]. In vitro study showed that in cultured 3T3-L1 adipocytes apoA-I upregulated the expression of adiponectin in phosphatidylinositol 3-kinase (PI3K)-dependent manner [6]. In brown adipocytes, apoA-I upregulated the expression of uncoupling protein 1, which participated in the control of energy expenditure, possibly involved Adenosine 5‘-monophosphate-activated protein kinase (AMPK) signaling activation [16]. It is so far generally accepted that the multiple effect of apoA-I are results of intracellular signaling cascade triggered either through a direct interaction between apoA-I and surface receptors such as ATP-binding cassette transporter A1 (ABCA1) and *β* subunit of ATP synthase *(β*-ATPase) [17–19] or through lipid raft disruption resulted from apoAI-ABCA1 complex induced cholesterol efflux [9, 10]. Although the phenomenon of apoA-I internalization has been observed in vascular cells such as macrophage and endothelium, the physiological significance remains undetermined. Besides, uptake and resecretion of apoA-I by adipocytes has only been demonstrated in recent years.

### Methods to study the recycling of ApoA-I by adipocytes

Studies in intracellular transport of lipoproteins demand specific and robust labeling of the target entity with minimal alteration of the investigated transport process by the labeling strategy. To achieve this goal, recombinant apoA-I needs to be constructed so that the modified apoA-I can bind with the labeling probe without affecting its main biological properties. Pulse-chase experiments of isotope- or fluorescence-labeled proteins are the usual approach used to gather dynamic information about apoA-I internalization and resecretion. Confocal studies of labeled apoA-I and labeled markers of specific organelles or proteins can give information about apoA-I localization and possible interaction with other proteins.

Fluorescent labeling was used to address the cellular uptake of apoA-I, which relies on labeling of a recombinant form of apoA-I with a fluorescent probe at a single and highly targeted site in the molecule without altering the main function of apoA-I (the ability to promote cholesterol efflux). Because human apoA-I ordinarily lacks a cysteine, which is necessary for a thiol-reactive probe (e.g. agent Alexa Fluor 546 C5-maleimide) to de attached, recombinant forms of apoA-I needs to be constructed. Cys residue needs to be introduced without affecting the ability of apoA-I to promote cholesterol efflux.

In humans, apoA-I is synthesized in the liver and intestinal cells as a non-glycosylated pre-pro-protein [20]. The 18 amino acid pre-segment is removed before the protein leaves the cell whilst the 6 amino acid pro-segment is cleaved during post secretion, leaving the mature 243 amino acid protein [21]. Since apoA-I is non-glycosylated, bacterial expression system such as *Escherichia coli* is usually the preferred option because eukaryotic systems (e.g. Chinese hamster ovary cell system, CHO) are generally more difficult to transfect than bacteria. Besides, once successfully transfected, CHO cell colonies must undergo an in-depth screening process to find stably transfected, high expression colonies. In addition, eukaryotic cells require a longer period of time for expression and a much higher level of maintenance than bacteria. However, if the pro-segment of apoA-I was removed, expression level in *Escherichia coli* would be very low unless one modifies the existing DNA sequence for the first 8 amino acid to a sequence in which bacterial codons are more readily available [22]. Therefore, fusion proteins containing a histidine tag which can be purified over a nickel chelating column have been created for the purpose of both expression and purification. Before using these expression products in experiments, the His tag sequence needs to be proteolytically cleaved away from the target sequence because it may adversely affect both structure and function of the apoA-I. Enteropeptides [23], factor Xa [24], and thrombin [25] are often used proteolytic systems and work well for many proteins. However, their use for apolipoprotein systems are limited by the fact that these proteases often cut at locations within the target protein in addition to the intended site engineered between the fusion and target proteins. In the case of apoA-I, this problem has been circumvented by lipidating the protein before cleavage, masking the secondary cleavage sites through a conformational change [26]. Another method to address this problem was IgA protease system. It was found that IgA protease from *Neisseria gonorrhoeae* did not cleave the wild type apoA-I sequence [27]. Therefore, adding an Igase site to the pET30 expression vector containing the apoA-I cDNA, IgA protease cleaved the His-tag without affecting the target apoA-I product [27]. As an alternative to protease-dependent removal of the N-terminal His-Tag, an acid labile Asp-Pro peptide bond was introduced into the apoA-I sequence between amino acids 2 and 3 of the wild type apoA-I sequence, which was achieved by mutating Glu2 to Asp, taking advantage of the presence of the Pro at position 3 in wild type apoA-I. Formic acid could efficiently cleave the apoA-I His-Tag at this acid labile peptide bond without exerting discernable effect on the apoA-I product, presenting as an efficient way for protein purification [28]. Fluorescent microscopy studies were then used to follow the cell biology of fluorescent labeled apoA-I.

Although fluorescent microscopy is well suited for measuring fluorescent apoA-I internalization, it gives little information on the fate of the label after internalization. Therefore, pulse-chase experiment through radiolabeled apoA-I could be used to study the fate of the internalized protein. Because nonspecific radioiodination of apoA-I can have significant effect on its structure and metabolism [29], endogenous labeling of wild-type apoA-I expressed in methionine auxotrophline line of bacteria (E. coli) by ^35^S-methionine is nowadays a commonly accepted method [12].

Although approaches based on the use of fluorescent labeled or radiolabeled apoA-I are highly valuable to study lipoprotein uptake, it is necessary to realize that there are some limitations due to unspecific labeling of cellular compartment caused by partial degradation of apoA-I followed by release of the small labeling probe. Besides, there is difficulty in distinguishing cellular incorporation of the protein from protein absorption to plasma membrane.

To solve the problem, a method was developed which could provide an unambiguous proof of apoA-I recycling by adipocytes through creating a functional recombinant apoA-I containing a phosphorylation site (pka-apoA-I), which could be recognized by the catalytic subunit of cAMP-dependent protein kinase (PKA) [15]. Because cellular uptake of the protein would allow its phosphorylation and the phosphorylated protein would be found in the cell culture medium if re-secreted, phosphorylated apoA-I in the medium was correlated to the extent of apoA-I recycling. pka-apoA-I construct was prepared by sub-cloning the full-length sequence of mature human apoA-I into a commercial vector that incorporated an N-terminal tag encoding for a six-His tag and a five amino acid recognition sequence (RRASV) for the catalytic subunit of PKA. Adipocytes were pre-incubated with ^32^P-phosphate to radiolabel the cellular pool of ATP. Phosphorylated apoA-I collected from the culture medium after different times of incubation with pka-apoA-I were subjected to SDS-PAGE, followed by coomassie blue staining and autoradiography to analyze the recycling of apoA-I by adipocytes [14, 15].

### Mechanism of ApoA-I uptake by adipocytes

#### Receptor mediated process

ApoA-I entering into adipocytes may be a receptor mediated process or the result of nonspecific endocytic membrane invagination. In line with several other cell types [12], a receptor mediated process was supported by the results that the content of phosphorylated pka-apoA-I in the culture medium, which represented the rate of apoA-I recycling, increased in response to increase in concentration of recombinant apoA-I (pka-apoA-I) and approached a plateau at concentrations higher than 75μg/ml. Besides, human apoA-I purified from plasma reduced apoA-I recycling in a dose dependent manner, indicating a competition between human and recombinant apoA-I for binding to a common receptor [14].

#### Clathrin

While LDL endocytosis occurs via clathrin-coated pits [30], it is unclear whether apoA-I internalization is clathrin dependent. It was shown that blocking clathrin-mediated endocytosis by its specific inhibitor monodansyl cadaverine (MDC) abolished apoA-I internalization into macrophage (RAW and THP-1 cell), suggesting uptake of apoA-I by macrophages is clathrin-dependent [13, 31]. However, considering heterogeneity may exist between different tissues and cell lines, further study need to be carried out to clarify if clathrin-pathway is involved in apoA-I uptake by adipocytes.

#### Caveolae

Caveolae, implicated in a variety of physiological process including cell signaling and endocytosis, are curved lipid raft regions rich in cholesterol and sphingolipids [32]. Caveolin-1, which is the main protein constituent of caveolae, has been shown to interact with ABCA1 and is involved in cholesterol efflux to apoA-I in several cell types [33]. Direct interaction of caveolin-1 with apoA-I has also been reported. Mouse embryonic fibroblasts (MEFs) derived from wild type animals were reported to have 2.6-fold more apoA-I binding sites than MEFs derived from caveolin-1 deficient animals. In addition, caveolin-1 binding with apoA-I was reported to target apoA-I for internalization [34]. Considering the remarkably abundant caveolae on the surface of adipocytes which accounts for 50 % of the surface area [32], it is speculated that caveolae may be involved in apoA-I uptake by adipocytes. Whether caveolae is pre-requisite for apoA-I internalization and whether the presence of caveolae accounts for a favorable environment for apoA-I internalization warrant further study.

#### ABCA1 independent process

Because apoA-I could directly bind with ABCA1 which rapidly shuttles between intracellular compartment and plasma membrane [35], whether apoA-I internalization is ABCA1 dependent has been explored. Previous studies which focused on macrophages observed co-localization of apoA-I and ABCA1 in endosomal compartment [36–38], supporting the idea that ABCA1-apoA-I complex were delivered to intracellular compartment where it received lipids and formed a nascent-HDL like particles that were subsequently secreted from the cell. However, a recent study carried out in adipocytes showed that Brefeldin A (BFA), which inhibited vesicular trafficking of ABCA1, didn’t affect apoA-I recycling by adipocytes, suggesting an ABCA1 independent way for apoA-I uptake may exist [14].

#### Mediated by *β*-ATPase

*β-*ATPase mainly presents in mitochondria membrane and is the terminal enzyme of the oxidative phosphorylation pathway. Unexpectedly, ectopic localization of *β-*ATPase on cell surface were found in several cells types including hepatocytes and adipocytes. What’s intriguing is that *β-*ATPase has been characterized as a receptor for apoA-I [15, 17, 39]. The binding of apoA-I to *β*-ATPase induced ATP hydrolysis and promoted extracellular ADP generation, which in turn stimulated intracellular signaling by activating purinergic receptors, which belong to the family of G-Protein-coupled receptors stimulated by extracellular nucleotides. In hepatocytes, it was demonstrated the interaction of the *β*-ATPase with apoA-I modulated HDL endocytosis [17, 40]. Niacin, which is used to raise plasma levels of HDL, has been shown to reduce the ectopic expression of the *β*-ATPase in hepatocytes and inhibit cellular uptake of HDL [41]. In adipocytes, *β*-ATPase has been proven to be involved in apoA-I uptake, evidenced by the fact that apoA-I recycling was blocked by a monoclonal antibody against the *β*-ATPase [15].

#### Regulation of apoA-I internalization through cAMP-PKA signaling

Previous studies revealed that the interaction between apoA-I and ABCA1 activated adenylate cyclase and increased the content of cAMP [42]. cAMP/protein kinase A (PKA) were reported to play an important role in regulating apoA-I/ABCA1-induced lipid translocation and removal. Pharmacologic modulation to decrease cAMP levels and PKA activity led to reduction in apoA-I-ABCA1 mediated cholesterol efflux [43, 44]. Conversely, interference to activate cAMP/PKA signaling elevated apoA-I-induced cholesterol efflux [44]. The molecular mechanisms may include: 1) regulation of ABCA1 expression by cAMP/PKA signaling at the transcriptional level [42], 2) phosphorylation of ABCA1 by PKA may result in altering the conformation of the protein to a more active state for lipid translocation across the cell membrane [45]. However, an interesting phenomenon was observed in the murine macrophages RAW 264 cell that cholesterol efflux to apoA-I was absent in the absence of cAMP but inducible by cAMP analogues 8-Br-cAMP [31, 46]. Coincidently, compared with cAMP treatment, cellular uptake and resecretion of apoA-I was apparently much less in the basal condition when cAMP is absent [31, 46]. Based on these interesting findings, it was queried was cellular internalization of apoA-I pre-requisite for apoA-I-induced cholesterol efflux or were they just two independent events subsequent to apoA-I binding with surface receptor? Based on previous studies carried out in macrophages, it is generally accepted that the contribution of internalized apoA-I to cholesterol efflux and HDL formation seems to be limited [12, 47].

In adipocytes, cAMP/PKA signaling was demonstrated to regulate apoA-I recycling. Stimulation with isoproterenol, which raised cAMP and activated PKA, accelerated recycling of apoA-I by 3 T3-L1 adipocytes. PKA inhibitor H89 inhibited the internalization and resecretion of apoA-I [14, 15]. The molecular mechanism underlying the regulatory effect of cAMP-PKA signaling on the recycling of apoA-I by adipocytes remains unclear. Whether PKA would regulate the binding ability of apoA-I with surface *β*-ATPase, which is involved in mediating apoA-I internalization, is not known. In addition, whether PKA may regulate the expression of *β*-ATPase or its translocation to the plasma membrane need to be further investigated.

### Physiological role of ApoA-I internalization in adipocytes

#### ApoA-I internalization and adipocyte cholesterol efflux

The physiological significance of apoA-I internalization in adipocytes is not known. As mentioned above, the mechanism of lipid-free apoA-I lipidation is not fully understood yet and whether apoA-I lipidation may require its internalization remains controversial. Some studies supported the idea that apoA-I lipidation mainly occurred at the cell surface. A model by Phillips proposed that ABCA1 shuttled phospholipids from the inner to extracellular surface of the plasma membrane, resulting in membrane bulges with high curvature that were sufficient to allow apoA-I penetration. Once apoA-I bound to ABCA1 on the cell surface and was lipidated, the N-terminus of apoA-I was unfolded and formed an unstable intermediate structure that was rapidly released from the cell [48, 49]. On the other hand, other studies reported that apoA-I internalization was involved in apoA-I induced cholesterol efflux. In previous reported studies, RAW cells were cholesterol loaded, incubated with apoA-I and treated with 8-Br-cAMP to stimulate the take up of apoA-I. After indicated period of treatment, apoA-I was depleted from the culture medium through washing. Significant cAMP-dependent [^3^H]-cholesterol efflux was detected during chase period even in the absence of exogenous apoA-I and HDL in the culture medium. Besides, the degree of cholesterol efflux was positively correlated to the extent of apoA-I internalization, indicating cholesterol efflux detected in this study was due to re-secretion of the internalized and lipidated apoA-I [31].

As the body’s largest pool of free cholesterol, adipose tissue was demonstrated to release cholesterol and contribute to HDL biogenesis [2, 3, 50]. Is apoA-I internalization involved in adipocytes cholesterol release? First of all, internalization of apoA-I is not indispensable for adipocytes cholesterol release because blocking apoA-I internalization does’t have a significant effect on cholesterol efflux [15]. However, it is worth noting that under basal condition cellular uptake of apoA-I is limited. Therefore, it may be more appropriate to consider the significance of apoA-I internalization under circumstances when the degree of apoA-I internalization is more prominent.

#### Intracellular cholesterol transport during cholesterol mobilization

It is interesting to note that both apoA-I internalization and adipocyte lipolysis are stimulated by PKA activation. Multiple evidence suggested that cholesterol and triglyceride homeostasis were coupled in adipocytes [51, 52], which led to the question what the role of apoA-I in intracellular cholesterol transport during lipid mobilization may be. Adipose is the body’s largest pool for free cholesterol. In adipocytes, <6 % cholesterol stored is in the esterified from [53, 54], which is different from other cell type [55]. Surface layer of lipid droplet was identified as primary intracellular storage site for free cholesterol within adipocytes [56]. It was shown in 3 T3-L1 adipocytes that under basal conditions cholesterol mobilization from adipocytes onto apoA-I was very limited despite induction of ABCA1 expression. However, under conditions of sustained lipolytic stimulation, a boost in the mobilization of adipocyte cholesterol was observed [52]. Besides, stimulation of lipolysis is accompanied by an increased cholesterol transport from surface of lipid droplet to plasma membrane (PM) where cholesterol can be removed [51]. Generally, there are vesicular and nonvesicular ways through which the highly hydrophobic free cholesterol can be transported from the lipid droplet pool to PM-pool. Cholesterol could be present in the membrane of intracellular vesicles for trafficking, which requires intact cytoskeleton and ATP to provide moving tracks and energy. Nonvesicular transport is mediated by diffusible carrier proteins, which have hydrophobic cavities to bind cholesterol and transport it across the aqueous cytosol [57, 58]. Cholesterol, together with phospholipids and proteins, were reported to be incorporated into cytosolic lipid protein particles (CLPP) which were HDL-like cytosolic lipid-protein complex with density of 1.09-1.16 g/ml and diameters of 17-18 nm [59]. In order to transport cholesterol to the target cellular compartment, the cholesterol transfer protein in CLPP needs to contain a specific membrane contact site. For example, intracellular transport from late endosome to mitochondria involves steroidogenic acute regulatory (StAR) protein on the mitochondria and cholesterol donating StAR-related lipid transfer (START) domain protein 3 (StARD3) on late endosomes [60, 61]. In the case of cholesterol transport from the ER (endoplasmic reticulum) to Golgi, cholesterol transport depends on the activity of oxysterol binding protein (OSBP) to create membrane contact sites between both organelles [62, 63]. What’s interesting is that caveolin-1, a critical protein found in membrane domain caveolae, has been found as protein constituent of CLPP. Since direct interaction of apoA-I with caveolin-1 has been confirmed at the cell surface [34], it would be interesting to study intracellular interaction of these two proteins and its possible role in intracellular cholesterol transport. It is speculated that intracellular apoA-I may be involved in CLPP formation, facilitating intracellular cholesterol transport to plasma membrane for further removal through efflux pathway. Therefore, apoA-I internalization and resecrestion by adipocytes may be accompanying process of cholesterol mobilization and represent an alternative pathway to maintain cholesterol homeostasis.

#### Relevance to obesity

Recently, the possible anti-obesity effect of apoA-I has triggered great interest. In mouse models, apoA-I transgenic mice had significant lower fat content than wild type mice after feeding with high fat diet for three months [16]. In other studies, daily administration of apoA-I mimetics D-4 F and L-4 F in high fat diet fed mice reduced weight gain and decreased obesity when compared with age-matched vehicle-treated obese mice [5, 64]. Several studies demonstrated that apoA-I may contribute to modulating body fat content by controlling the extent of lipolysis [5, 65]. Recent study showed that the naturally occurring apoA-I variant Milano, which contains a single point mutation that lead to an Arg (173) Cys substitution, reduced fat mass through stimulation of lipolysis. Similar to apoA-I WT, the anti-obesity effect of Milano was independent of ABCA1 nor the canonical cAMP/PKA signaling pathway [66]. Interestingly, the Milano-stimulated lipolysis was much greater compared with apoA-I WT [66]. Since apoA-I Milano was demonstrated to be more efficient in modulating cholesterol efflux than apoA-I WT [67], this finding further supported the notion that cholesterol mobilization was coupled with lipolysis. It would be interesting to further investigate the significance of adipocytes apoA-I recycling in obesity.

Ectopic expression of *β*-ATPase on the plasma membrane of adipocytes was demonstrated to be involved in mediating the endocytosis and re-secretion of apoA-I [15]. Interestingly, the ectopic expression of *β*-ATPase was found to be increased during adipogenesis [68]. However, the physiological significance of *β*-ATPase in adipogenesis is not known. Our preliminary study observed that apoA-I could regulate adipocyte differentiation with a mechanism which is not clear. Therefore, further studies should be carried out to investigate apoA-I recycling at different stage of adipogenesis. Whether apoA-I recycling is involved in adipogenesis is an interesting question for further exploration.

#### Transport of cellular miRNA

It has been reported recently that HDL can transport microRNAs [69], which are short non-coding regulatory RNAs that modulate biological homeostasis by controlling gene expression through mRNA target and translational repression. Circulating miRNAs can be transported from donor cells to recipient cells and are viewed as a new class of biomarkers for a diverse set of diseases [70]. As the main protein component of HDL, apoA-I has been used for the systemic and specific delivery of small interfering RNA (siRNA) to hepatocytes in animal models [71]. It is interesting to hypothesize that apoA-I might acquire microRNAs during intracellular trafficking in adipocytes and deliver regulatory information to recipient cells when it is re-secreted into the plasma.

### Summary

To sum up, uptake and resecretion of apoA-I by adipocytes were detected by using different apoA-I-labeling methods. Current studies on the mechanisms of apoA-I uptake by adipocytes support that it is a receptor mediated process which is ABCA1 independent but involves *β*-ATPase ectopically expressed on the plasma membrane. Besides, cAMP/PKA signaling regulates recycling of apoA-I by adipoctes, with the underlying mechanisms remain unclear. Whether apoA-I internalization is a clathrin and caveolae dependent process needs to be further studied. Although apoA-I recycling by adipocytes does not seem to play a major role in regulating adipose cholesterol metabolism under physiological conditions, it is likely that apoA-I recycling becomes important in pathological conditions when cholesterol mobilization is stimulated. Because cholesterol and TG metabolism are coupled in adipose tissue and recent recognition of the relationship between apoA-I and obesity, the physiological relevance of apoA-I recycling to obesity and adipogenesis are interesting questions to be answered. During intracellular transport, apoA-I may function as a vehicle through interaction with other proteins. The cargo is not restricted to intracellular cholesterol but may also include miRNAs, which modulate gene expression of target cells when apoA-I-miRNAs complex are re-secreted into the circulation by adipocytes.

## Abbreviations

ABCA1: ATP-binding cassette transporter A1
AMPK: adenosine 5‘-monophosphate-activated protein kinase
apoA-I: apoliprotein A-I
Asp: aspartate
CLPP: cytosolic lipid protein particles
Cys: cysteine
ER: endoplasmic reticulum
MCP-1: monocyte chemotactic protein-1
MDC: monodansyl cadaverine
MEFs: mouse embryonic fibroblast
NADPH oxidase: nicotinamide adenine dinucleotide phosphate-oxidase
OSBP: oxysterol binding protein
PI3K: phosphatidylinositol 3-kinase
PKA: protein kinase A
pka-apoA-I: recombinant apoA-I containing a phosphorylation site
PM: plasma membrane
Pro: proline
ROS: reactive oxygen species
SSA3: serum amyloid A3
StAR: steroidogenic acute regulatory
StARD3: START domain protein 3
START: StAR-related lipid transfer
*β*-ATPase: *β* subunit of ATP synthase
